# Characteristics of peripapillary retinal nerve fiber layer atrophy in glaucoma, optic nerve sheath meningioma, and sphenoid wing meningioma

**DOI:** 10.1007/s00417-021-05354-2

**Published:** 2021-09-23

**Authors:** Felix Tonagel, Helmut Wilhelm, Carina Kelbsch, Paul Richter

**Affiliations:** Centre for Ophthalmology, University of Tuebingen, Elfriede-Aulhorn-Str. 7, 72076 Tuebingen, Germany

**Keywords:** Peripapillary retinal nerve fiber layer, RNFL, Optic atrophy, Glaucoma, Meningioma, POAG, ONSM, SWM

## Abstract

**Background/objectives:**

The correct classification of a slowly progressing optic atrophy can be challenging. The aim of this work was to find out if the characteristics of peripapillary retinal nerve fiber layer (RNFL) thickness loss differ between open angle glaucoma (POAG), optic nerve sheath meningioma (ONSM), and sphenoid wing meningioma (SWM).

**Methods:**

A total of 45 patients with POAG, ONSM, and SWM were included in the retrospective study. The peripapillary RNFL thickness measured by spectral-domain optical coherence tomography was analyzed using the Heidelberg Engineering glaucoma module^©^.

**Results:**

Each group consisted of 15 patients. The temporal sector of the RNFL thickness showed a median decrease of − 17 µm in glaucoma patients (range + 6/–34 µm), − 43 µm in ONSM (range − 19/ − 52 µm), and − 44 µm in SWM patients (range − 25/ − 52 µm). The RNFL thickness of the temporal sector of glaucoma patients differed significantly from the other groups (*p* < 0.001). All other sectors showed no significant difference between the 3 groups.

**Conclusion:**

The peripapillary RNFL thickness of the temporal sector of patients with beginning to moderate POAG is usually inside normal limits or borderline. In contrast, patients with ONSM and SWM are much more likely to show a considerable reduction in RNFL thickness of the temporal sector. RNFL thickness of the temporal sector marked outside normal limits occurred exclusively in meningioma patients. Considering the presence of this condition as a predictor for meningioma, sensitivity and specificity were 0.8 and 1.0, respectively. In patients with significant reduction in RNFL thickness of the temporal sector, magnetic resonance imaging of the head should be considered to rule out compression of the optic nerves.

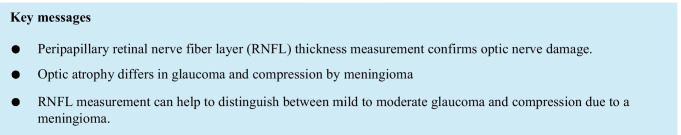

## Introduction

Various diseases may be associated with a slowly progressive degeneration of retinal ganglion cell (RGC) axons. This can be detected by measuring the peripapillary retinal nerve fiber layer (RNFL) via optical coherence tomography (OCT). Since RGC loss is irreversible and can lead to visual field defects and vision loss including blindness, it is important to make the appropriate diagnosis as early as possible. Possible underlying causes are glaucoma [1] or compression of the optic nerve by a tumor [2]. The correct classification of the cause of a slowly progressing optic atrophy can be challenging. Glaucoma is a relatively common disease with a prevalence in the population aged 40 years or older of 35% [3]. Compressive optic neuropathy is much rarer with an incidence of about 4 per 100,000 [4]. Nevertheless, these diseases are similar both in the painless loss of RGC axons and in the possible symptoms such as visual field defects and visual impairment. In rare cases, glaucoma and compressive optic neuropathy even occur together. However, the therapy is fundamentally different, so early diagnosis is important to avoid further permanent optic nerve damage. The aim of this work was to find out if the characteristics of RNFL thickness loss differ between open angle glaucoma (POAG), optic nerve sheath meningioma (ONSM), and sphenoid wing meningioma (SWM) compressing the optic nerve. We chose these three entities because they all may lead to painless and slowly progressive optic neuropathy. The aim of this study was to investigate if it was possible to distinguish glaucomatous and compressive optic neuropathy by OCT alone.

## Materials/subjects and methods

A total of 45 patients with primary open angle glaucoma (POAG), optic nerve sheath meningioma (ONSM), and sphenoid wing meningioma (SWM) of the inner and outer ridge compressing the optic nerve were included in the retrospective study. Patient recruitment took place consecutively. If both eyes were affected by the disease, one eye was randomly selected and evaluated. ONSM were diagnosed in all patients by multiple MRI scans. In addition, DOMITATE or DOTATOC-PET-CT were performed in 10 of the 15 ONSM-patients. All SWM were examined histologically. The peripapillary RNFL thickness measured by spectral-domain optical coherence tomography (SD-OCT) was analyzed. The Heidelberg Engineering glaucoma module^©^ was used, which performs a circular peripapillary measurement of the RNFL with 3.5 mm diameter. The RNFL thickness is calculated for 6 different sectors and total thickness and divided into the groups “inside normal limits,” “borderline,” and “outside normal limits” automatically. We used this classification and also analyzed the deviation from the average RNFL thickness of normal eyes of each sector measured in microns. Values of normal eyes were taken from the Spectralis reference database “European Descent 2009” that is implemented in the Heidelberg Engineering glaucoma module^©^. Since normal nerve fiber layer thickness differs between sectors, the absolute RNFL losses required for the classification “borderline,” “outside,” and “inside normal limits” differ depending on the sector considered.

To be included in the study, one of the abovementioned diseases had to be present and at least one sector of the RNFL thickness had to be classified as “borderline” or “outside normal limits.” More than 4 external sectors classified “outside normal limits” as well as funduscopically visible papilledema led to exclusion from the study. Patients with other diseases of the retina or optic nerves were also excluded from the study. The OCT scans were evaluated by an experienced ophthalmologist for technically correct performance, in particular for correct placement of the examination ring in relation to the optic disc. Only patients with correctly performed OCT examination were included in the study.

All analyses were performed using JMP® 14.2.0 statistical software (SAS Institute, Cary, NC, USA). Continuous data were summarized with the median and range; categorical data are reported as numbers and percentages. To compare continuous unpaired data between groups, the two-sided two-sample *t* test was used. Categorical data were tested using the chi-squared test. The analysis of variance as a function of the 3 etiologies was carried out using the f-test. A *P* value of less than 0.05 was considered statistically significant.

## Results

**Fig. 1:**
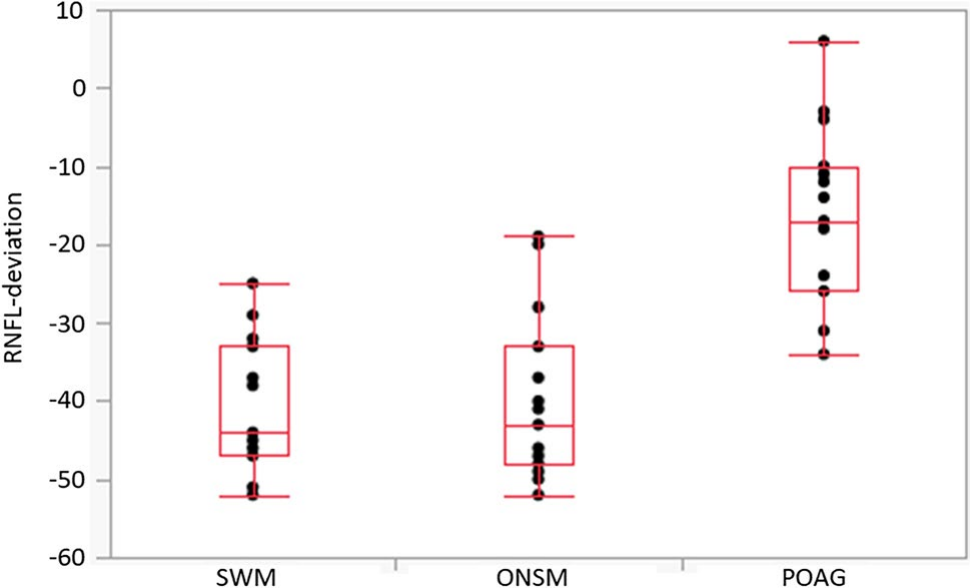
Shown is the deviation of the RNFL thickness of the temporal sector from the mean layer thickness of healthy eyes according to the Spectralis reference database “European Descent 2009”

Each group consisted of 15 patients. Median age of POAG patients was 64 years (range 40/85), 60% females; the median decimal visual acuity was 1.0 (range 0.5/1.0, Table 1). In the ONSM group, the median age was 46 years (range 9–68), 80% females; the median decimal visual acuity was 0.63 (range 0–1.0). Median age in the SWM group was 49 years (range 35/78), 67% females; the median decimal visual acuity was 0.4 (range 0.02/1.0). Total peripapillary RNFL thickness was outside normal limits in 80% (12 out of 15) in the POAG and SWM group, respectively, and 73% (11 out of 15) in the ONSM group (Table 2). All groups showed frequent damage to the temporal superior and temporal inferior RNFL thickness (Tables 1 and 2). The temporal sector of RNFL thickness showed a median decrease of − 17 µm in glaucoma patients (range + 6/– 34 µm), − 43 µm in ONSM patients (range − 19/ − 52 µm), and − 44 µm in SWM patients (range − 25/ − 52 µm). The RNFL thickness of the temporal sector of glaucoma patients differed significantly from the other groups (*p* < 0.001, Fig. 1). In the cohort studied, RNFL thickness of the temporal sector marked “outside normal limits” occurred exclusively in meningioma patients. Considering the presence of this condition as a predictor for meningioma, sensitivity and specificity are 80% and 100%, respectively; the positive predictive value is 100%, and the negative predictive value 71.4%. All other sectors showed no significant difference between the 3 groups: The nasal inferior and nasal sectors had only a slight reduction of RNFL thickness in all groups: The nasal superior, nasal inferior, and nasal sectors were reduced by − 29 µm, − 34 µm, and − 15 µm for POAG (range + 3.0/ − 52 µm, + 3/ − 75 µm, and + 3/ − 45 µm), − 36 µm, − 19 µm, and − 20 µm for ONSM (range + 29/ − 62 µm, + 28/ − 46 µm, and + 32/ − 37 µm), and − 39 µm, − 23 µm, and − 21 µm for SWM (range + 32/ − 65 µm, + 27/ − 53 µm, and + 8/ − 28 µm).

**Table 1:**
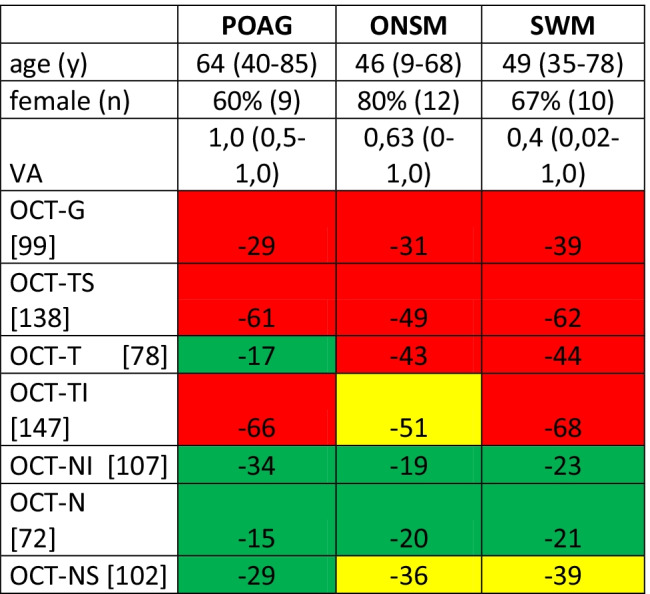
Median deviation of standard RNFL thickness, visual acuity, gender, and age of the disease groups

*RNFL* rating according to Heidelberg engineering Spectralis glaucoma module: red outside normal limits, yellow borderline, green within normal limits. Indicated is the deviation from the average of normal eyes. Values of normal eyes are taken from the Spectralis reference database and given in square brackets

*OCT-G* total RNFL-thickness, *OCT-TS* temporal-superior RNFL-thickness, *OCT-T* temporal RNFL thickness, *OCT-TI* temporal-inferior RNFL-thickness, *OCT-NI* nasal-inferior RNFL-thickness, *OCT-N* nasal RNFL-thickness, *OCT-NS* nasal-superior RNFL-thickness

**Table 2:**
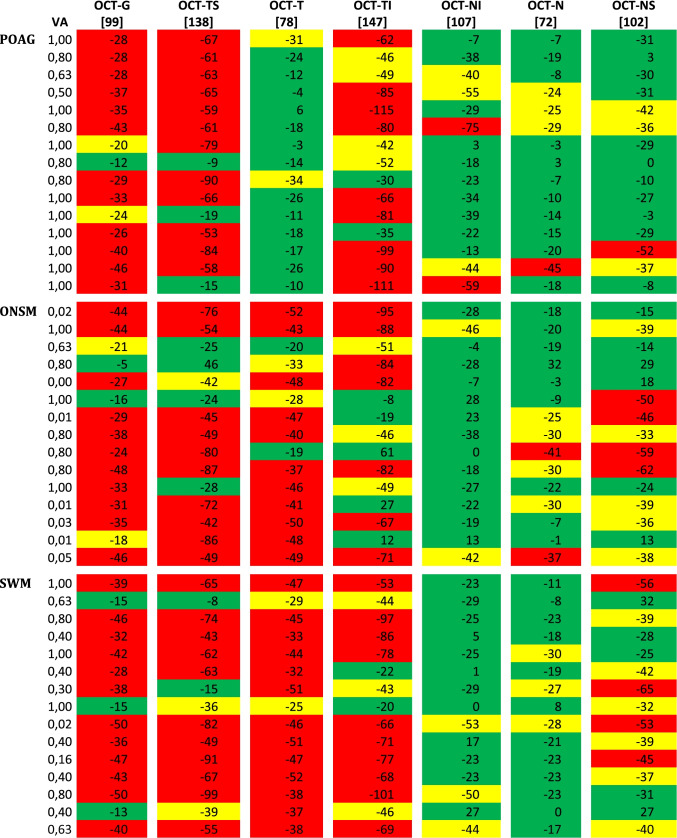
Deviation of standard RNFL thickness and visual acuity for the underlying diseases

*RNFL* rating according to Heidelberg engineering Spectralis glaucoma module: red outside normal limits, yellow borderline, green within normal limits. Indicated is the deviation from the average of normal eyes. Values of normal eyes are taken from the Spectralis reference database and given in square brackets

*OCT-G* total RNFL-thickness, *OCT-TS* temporal-superior RNFL-thickness, *OCT-T* temporal RNFL thickness, *OCT-TI* temporal-inferior RNFL-thickness, *OCT-NI* nasal-inferior RNFL-thickness, *OCT-N* nasal RNFL-thickness, *OCT-NS* nasal-superior RNFL-thickness

## Discussion

Our study showed that peripapillary nerve fiber layer thickness in the temporal sector allowed to differentiate between glaucomatous and compressive optic neuropathy with a sensitivity of 80% and specificity of 100%. To obtain uniform patient groups, we decided to select only sphenoid wing meningiomas and optic nerve sheath meningiomas for the optic nerve compression groups. It is likely that other tumor types lead to similar RNFL damage. The knowledge gained from this study is not intended to replace other tests such as the examination of the visual field but can help to make the correct diagnosis more quickly in the case of inconclusive test results.

Leung et al. examined RNFL thickness of a 6 × 6-mm^2^ peripapillary region of glaucoma patients and found that RNFL loss was most common inferotemporal and superotemporal, consistent with the results of our study [5]. Furthermore, our results indicate in contrast to a relative sparing of the temporal RNFL sector in glaucoma that ONSM and SWM regularly cause temporal damage to the RNFL. To our knowledge, the damage pattern of ONSM and SWM has not yet been investigated. Thus, the works of Miller [6] and Douglas et al. [7] establish a relationship between ONSM and optic atrophy, but do not analyze the damage pattern more precisely. Interlandi et al. reported [8] on a reduction of temporal RNFL thickness in a single-case report of ONSM.

A possible source of error is incorrect positioning of the scan ring during the first OCT examination of a patient, since a horizontally misplaced scan ring can simulate a RNFL thickness loss of the temporal sector. It is therefore necessary to check the correct placement of the OCT scan ring for each patient. Another source of error would be if the total RNFL thickness loss of the different groups were significantly different. This has been avoided by the abovementioned definition of the inclusion and exclusion criteria, which allowed only mild to moderate optic nerve damage. One limitation of the study results is that they are not applicable to all degrees of optic nerve damage: If the optic atrophy is very severe, it may no longer be possible to differentiate between the disease entities. For this reason, we only included patients with mild to moderate peripapillary RNFL thickness loss in the study. Furthermore, a RNFL measurement of ONSM patients with papilledema does not give meaningful results. Although eyes with clinically visible papilledema were not included in the study, it is noticeable that positive deviations were found most frequently in the ONSM group, i.e., the corresponding thickness was greater than would be expected in normal eyes. Such subclinical papilledema in ONSM patients may have influenced the analysis of these sectors.

In summary, the peripapillary RNFL thickness of the temporal sector of patients with beginning to moderate POAG is usually inside normal limits or borderline at most. In contrast, patients with ONSM and SWM seem to be much more likely to show a considerable reduction in RNFL thickness of the temporal sector. In the cohort studied, RNFL thickness of the temporal sector marked “outside normal limits” occurred exclusively in meningioma patients. Considering the presence of this condition as a predictor for meningioma, the positive predictive value is 100% in our cohort. The particular value of this observation lies in the high probability of predicting a meningioma in patients with slowly increasing RNFL thickness loss. This observation can help to distinguish between mild to moderate glaucoma and ONSM/SWM or even patients with ONSM/SWM in addition to a known glaucoma. In the case of significant reduction in RNFL thickness of the temporal sector, magnetic resonance imaging (MRI) of the head should be considered to rule out compression of the optic nerve.

## Data Availability

All data used are included in the publication.
